# Functional outcomes from a head-to-head, randomized, double-blind trial of lisdexamfetamine dimesylate and atomoxetine in children and adolescents with attention-deficit/hyperactivity disorder and an inadequate response to methylphenidate

**DOI:** 10.1007/s00787-015-0718-0

**Published:** 2015-05-22

**Authors:** Peter Nagy, Alexander Häge, David R. Coghill, Beatriz Caballero, Ben Adeyi, Colleen S. Anderson, Vanja Sikirica, Esther Cardo

**Affiliations:** Vadaskert Child and Adolescent Psychiatry Hospital and Outpatient Clinic, Budapest, Hungary; Paediatric Psychopharmacology, Department of Child and Adolescent Psychiatry and Psychotherapy, Central Institute of Mental Health, Medical Faculty Mannheim, University of Heidelberg, Mannheim, Germany; Division of Neuroscience, University of Dundee, Dundee, UK; Shire, Eysins, Switzerland; Shire, Wayne, PA USA; Neuropaediatric Unit, Son Llàtzer Hospital and Research Institute on Health Sciences, University of the Balearic Islands, Palma De Mallorca, Spain

**Keywords:** Atomoxetine, Attention-deficit/hyperactivity disorder, Functional impairment, Lisdexamfetamine dimesylate, Weiss Functional Impairment Rating Scale-Parent Report

## Abstract

Attention-deficit/hyperactivity disorder (ADHD) is associated with functional impairments in multiple domains of patients’ lives. A secondary objective of this randomized, active-controlled, head-to-head, double-blind, dose-optimized clinical trial was to compare the effects of lisdexamfetamine dimesylate (LDX) and atomoxetine (ATX) on functional impairment in children and adolescents with ADHD. Patients aged 6–17 years with an ADHD Rating Scale IV total score ≥28 and an inadequate response to methylphenidate treatment (judged by investigators) were randomized (1:1) to once-daily LDX or ATX for 9 weeks. Parents/guardians completed the Weiss Functional Impairment Rating Scale-Parent Report (WFIRS-P) at baseline and at week 9 or early termination. *p* values were nominal and not corrected for multiple comparisons. Of 267 randomized patients, 200 completed the study (LDX 99, ATX 101). At baseline, mean WFIRS-P total score in the LDX group was 0.95 [standard deviation (SD) 0.474; 95 % confidence interval (CI) 0.87, 1.03] and in the ATX group was 0.91 (0.513; 0.82, 1.00). Scores in all WFIRS-P domains improved from baseline to endpoint in both groups, with least-squares mean changes in total score of −0.35 (95 % CI −0.42, −0.29) for LDX and −0.27 (−0.33, −0.20) for ATX. The difference between LDX and ATX was statistically significant (*p* < 0.05) for the Learning and School (effect size of LDX vs ATX, 0.43) and Social Activities (0.34) domains and for total score (0.27). Both treatments reduced functional impairment in children and adolescents with ADHD; LDX was statistically significantly more effective than ATX in two of six domains and in total score.

**Electronic supplementary material:**

The online version of this article (doi:10.1007/s00787-015-0718-0) contains supplementary material, which is available to authorized users.

## Introduction

Attention-deficit/hyperactivity disorder (ADHD) is characterized by the core symptoms of inattention, hyperactivity, and impulsivity. ADHD is also associated with substantial functional impairments that can affect social, academic, and occupational activities throughout life [6]. Indeed, the Diagnostic and Statistical Manual of Mental Disorders, 4th Edition, Text Revision (DSM-IV-TR) [3], the recent 5th edition of the DSM (DSM-5) [2], and the International Classification of Diseases, 10th revision [43] all specify evidence of functional impairment as a diagnostic criterion for ADHD. The impact of such impairment on a patient’s daily life commonly provides the motivation to seek medical treatment [37]. ADHD treatment should, therefore, aim not only to improve symptoms but also to reduce functional impairment. This is reflected in European regulatory guidance for clinical trials of ADHD medications, which state that such trials should include a functional outcome measure [30].

Lisdexamfetamine dimesylate (LDX) is a long-acting prodrug psychostimulant. Clinical trials in children, adolescents, and adults have shown LDX to be effective in the treatment of ADHD and to have a tolerability profile consistent with that of psychostimulant therapy [7, 8, 22, 31]. LDX is approved as a first-line treatment for ADHD in the USA, Canada, Brazil, and Australia and is the first long-acting amfetamine-based medication to be approved in Europe, where it is licensed in select countries for the treatment of children and adolescents with ADHD when response to previous methylphenidate (MPH) treatment is considered clinically inadequate by the supervising specialist.

Study SPD489-317 (ClinicalTrials.gov NCT01106430) was a 9-week, head-to-head, randomized, double-blind trial comparing LDX with atomoxetine (ATX), a non-stimulant noradrenaline reuptake inhibitor [27]. The study was conducted in a population of children and adolescents with ADHD in Europe and North America who were judged to have responded inadequately to MPH therapy (defined in ‘Methods’). The primary efficacy outcome was the time to clinical response [defined as a Clinical Global Impressions-Improvement (CGI-I) score of 1 (very much improved) or 2 (much improved)]. This was significantly shorter for patients receiving LDX than for those receiving ATX [12.0 days (95 % confidence interval (CI) 8.0, 16.0) vs 21.0 days (15.0, 23.0); *p* = 0.001] [27].

Secondary efficacy outcomes revealed that significantly higher proportions of patients in the LDX group exhibited a clinical response (CGI-I score of 1 or 2) compared with those in the ATX group and that patients receiving LDX showed significantly greater improvements in ADHD symptoms (assessed using the ADHD Rating Scale IV [ADHD-RS-IV]) than those receiving ATX, at every study visit in weeks 1–9 [27]. Assessment of these outcomes used the ‘last observation carried forward’ approach to missing data. The safety and tolerability profiles of both treatments were consistent with findings from previous clinical trials [27].

Here, we report the effects of LDX and ATX treatment on functioning in study SPD489-317, as measured using a disorder-specific instrument, the Weiss Functional Impairment Rating Scale-Parent Report (WFIRS-P) [29]. This was a pre-specified secondary objective of study SPD489-317.

## Methods

This double-blind, randomized, active-controlled, parallel-group clinical trial was conducted between June 2010 and July 2012 at 51 sites in Europe, the USA, and Canada. The protocol was approved by an institutional review board, an independent ethics committee, or a regulatory agency at each centre, and the study was conducted in accordance with the International Conference on Harmonisation of Good Clinical Practice guideline and with local ethical and legal requirements. Full details of the study design have been published previously [27].

### Study population

Patients aged 6–17 years were eligible for enrolment if they met the DSM-IV-TR criteria for a primary diagnosis of ADHD, had a baseline ADHD-RS-IV total score of 28 or higher (indicating at least moderately severe symptoms), and had previously experienced (or were experiencing) an inadequate response to MPH treatment. ‘Inadequate response’ was judged by investigators and included, but was not limited to: variable or incomplete symptom control; inadequate duration of action; and the potential (in the investigator’s opinion) for the patient to benefit clinically from an alternative to MPH treatment. Patients were excluded if they had been exposed to amfetamine or ATX previously, if they had experienced intolerable side effects with previous MPH treatment, or if their symptoms were well controlled with acceptable tolerability on their current ADHD medication. Patients were also excluded if they had failed to respond to more than one previous course of MPH (defined as worsened, unchanged, or minimally improved symptoms) or if they had previously been treated with more than one formulation of MPH [except short-term (≤4 weeks) dose titration with immediate-release MPH, provided they experienced an adequate response]. Furthermore, patients were excluded if they had a comorbid psychiatric diagnosis with significant symptoms or other symptomatic manifestations (e.g. agitated states, marked anxiety, or tension) that contraindicated treatment with LDX or ATX in the opinion of the investigator. Patients with a conduct disorder were also excluded, but oppositional defiant disorder was not exclusionary.

### Study design

After discontinuing any previous psychoactive medication for a 7-day washout period, patients were randomized in a 1:1 ratio to receive once-daily treatment with LDX or ATX for 9 weeks. Doses of LDX and ATX were adjusted at weekly intervals over a 4-week period until an ‘acceptable’ response was achieved; this was defined as a reduction of at least 30 % in ADHD-RS-IV total score from baseline and a CGI-I score of 1 or 2 (very much improved or much improved) with tolerable side effects [27, 28, 33]. Patients in the LDX group initially received 30 mg/day, and if required, the dose was titrated to 50 mg/day and then to 70 mg/day. Patients in the ATX group weighing 70 kg or more initially received 40 mg/day, and if required, the dose was titrated to 80 mg/day and then to 100 mg/day. Patients in the ATX group weighing less than 70 kg initially received approximately 0.5 mg/kg/day, and if required, the dose was titrated to a final target dose of 1.2 mg/kg/day, with a maximum permitted dose of 1.4 mg/kg/day.

### Functional impairment assessments

Change in functional impairment, assessed using the WFIRS-P, was a secondary efficacy outcome of the study (the primary efficacy and safety outcomes have been published previously [27]). The WFIRS-P was completed by each patient’s parents or legal guardians at baseline (week 0) and either at week 9 or at an early termination visit attended by patients who withdrew from the study.

The WFIRS-P was designed to provide a disorder-specific measure of functioning in children and adolescents with ADHD and has been shown to have good internal consistency and moderate convergent validity with other instruments [29]. The questionnaire comprises 50 items, grouped into six domains (Family, Learning and School, Life Skills, Child’s Self-Concept, Social Activities, and Risky Activities). Each item relates to the previous month and is scored on a four-point Likert scale (0 = never or not at all; 1 = sometimes or somewhat; 2 = often or much; 3 = very often or very much) or recorded as not applicable [29]. This study used an early version of the WFIRS-P [41]; the current version refers to the Learning and School domain as the School domain [40]. Higher WFIRS-P scores indicate more severe functional impairment.

Data have not yet been published on the minimum changes in WFIRS-P scores from baseline to endpoint within a treatment group that correspond to clinically relevant improvements in functional impairment in patients with ADHD. One-half the standard deviation (SD) at baseline [34, 44] has been recommended as a value for the minimum clinically important difference in WFIRS-P scores by the developers of the instrument and has been used in previous studies [32]. To our knowledge, no data exist on minimum clinically important differences between two active medications in improvements in WFIRS-P scores.

### Statistical analysis

Sample size was calculated as previously described and was based on the primary efficacy outcome of time to clinical response (and not on any secondary outcome) [27]. WFIRS-P data were analysed for the Full Analysis Set, which comprised all patients who were randomized and received at least one dose of study medication, and which was based on the intention-to-treat principle. Baseline and week 9 scores were the observed values; endpoint was defined as the last on-treatment, post-baseline visit with a valid WFIRS-P assessment (endpoint is therefore equivalent to ‘last observation carried forward’ at week 9).

Changes in WFIRS-P total or domain scores from baseline to week 9 and endpoint were analysed using an analysis of covariance (ANCOVA) model with treatment group and country as fixed effects and baseline score as a covariate. The model did not include interaction terms. A formal statistical comparison between LDX and ATX for the least-squares (LS) mean change in WFIRS-P total and domain scores from baseline to endpoint was pre-specified in the study protocol. A formal statistical analysis of the change from baseline to week 9 or endpoint within each treatment group was not pre-specified, but was performed ad hoc, and was not therefore protected against type I error. No adjustment of *p* values for multiple comparisons was pre-specified or performed for secondary efficacy variables; *p* values less than 0.05 should therefore be regarded as representing nominal statistical significance. Effect sizes were calculated as the difference in LS mean change in scores from baseline between the LDX and ATX groups divided by the root mean square error obtained from the ANCOVA model. Effect sizes of 0.2, 0.5, and 0.8 are considered to indicate small, medium, and large differences between treatment groups, respectively, and are more often used for comparisons of an active drug versus placebo than of one active drug versus another [25].

## Results

### Patient disposition and baseline characteristics

Patients (*n* = 267) were enrolled at 51 study sites in the USA (*n* = 138), Germany (*n* = 42), Canada (*n* = 35), Spain (*n* = 22) Hungary (*n* = 20), Sweden (*n* = 6), Belgium (*n* = 2), Italy (*n* = 1), and Poland (*n* = 1). Of 267 patients randomized (LDX, *n* = 133; ATX, *n* = 134), 262 received at least one dose of study drug and were included in the Full Analysis Set (LDX, *n* = 127; ATX, *n* = 135). Of these, 200 (74.9 %) completed the study (LDX, *n* = 99; ATX, *n* = 101). Based on the intention-to-treat principle, one patient who was randomized to ATX but received LDX in error was included in the ATX group in the Full Analysis Set. The most common reasons for discontinuation in the LDX group were adverse events (*n* = 8; 6.0 %) and withdrawal by the patient (*n* = 8; 6.0 %); the most common reasons in the ATX group were lack of efficacy (*n* = 13; 9.7 %) and adverse events (*n* = 10; 7.5 %).

Baseline demographic characteristics were similar in both treatment groups [27]. The mean age of patients at baseline was 10.6 years (SD 2.93); 74.0 % of patients were children (aged 6–12 years), and 75.2 % were male. In both treatment groups, the most frequently reported reason for an inadequate response to previous MPH treatment was lack of efficacy [LDX 96/127 (75.6 %), ATX 106/135 (78.5 %)]. The mean optimal dose during the dose-maintenance phase (the dose that was dispensed from visit 4) was 52.5 mg/day (SD, 16.10) in the LDX group [28/128 (21.9 %), 36/128 (28.1 %), and 41/128 (32.0 %) patients received 30, 50, and 70 mg/day, respectively] and 40.2 mg/day (20.05) in the ATX group [of patients weighing <70 kg, 15/134 (11.2 %) and 95/134 (70.9 %) received 0.5 and 1.2 mg/kg/day, respectively; of patients weighing ≥70 kg, 2/134 (1.5 %), 1/134 (0.7 %), and 4/134 (3.0 %) patients received 40, 80, and 100 mg/day, respectively] [27].

### WFIRS-P scores at baseline

At baseline, mean WFIRS-P total scores and scores in each individual domain were similar across both treatment groups (Table 1; Figure S1). Mean WFIRS-P total scores were 0.95 (SD 0.474; 95 % CI 0.87, 1.03) in the LDX group and 0.91 (SD 0.513; 95 % CI 0.82, 1.00) in the ATX group (Table 1). Baseline data were not tested statistically for equivalence between treatment groups, but, in all domains and in total score, the mean score in the LDX group lay within the 95 % CI of the mean in the ATX group and vice versa (Figure S1).

**Table 1:** WFIRS-P total and domain scores at baseline, week 9, and endpoint, mean (SD)

	Baseline		Week 9		Endpoint	
	LDX (124 ≤ *n* ≤ 127)	ATX (128 ≤ *n* ≤ 135)	LDX (92 ≤ *n* ≤ 95)	ATX (93 ≤ *n* ≤ 97)	LDX (102 ≤ *n* ≤ 107)	ATX (110 ≤ *n* ≤ 114)
Family	1.18 (0.733)	1.11 (0.817)	0.66 (0.562)	0.75 (0.635)	0.71 (0.607)	0.80 (0.684)
Learning and school	1.20 (0.657)	1.19 (0.671)	0.54 (0.444)	0.69 (0.551)	0.56 (0.450)	0.72 (0.553)
Life skills	1.07 (0.496)	1.02 (0.571)	0.70 (0.447)	0.66 (0.450)	0.73 (0.472)	0.69 (0.483)
Child’s self-concept	0.83 (0.807)	0.72 (0.830)	0.41 (0.545)	0.39 (0.520)	0.45 (0.583)	0.44 (0.652)
Social activities	0.82 (0.663)	0.83 (0.702)	0.43 (0.427)	0.61 (0.530)	0.46 (0.468)	0.61 (0.544)
Risky activities	0.44 (0.434)	0.39 (0.376)	0.22 (0.228)	0.26 (0.257)	0.23 (0.244)	0.27 (0.260)
Total	0.95 (0.474)	0.91 (0.513)	0.51 (0.308)	0.59 (0.401)	0.54 (0.333)	0.62 (0.412)

Data are presented as mean (standard deviation). Higher scores indicate greater impairment. Baseline and week 9 scores are based on observed values. Endpoint was defined as the last on-treatment, post-baseline visit with a valid assessment. Numbers of observations (*n*) were in the ranges indicated, depending on domain. These data were not analysed statistically

*ATX* atomoxetine, *LDX* lisdexamfetamine dimesylate, *n* number of observations, *WFIRS-P* Weiss Functional Impairment Rating Scale-Parent Report

In both groups, the highest mean scores (indicating the greatest degree of impairment) were seen in the Family domain [LDX 1.18 (SD 0.733), ATX 1.11 (0.817)] and the Learning and School domain [LDX 1.20 (SD 0.657), ATX 1.19 (0.671)] (Table 1).

### Difference between LDX and ATX groups in change in WFIRS-P scores from baseline to endpoint

**Fig. 1:**
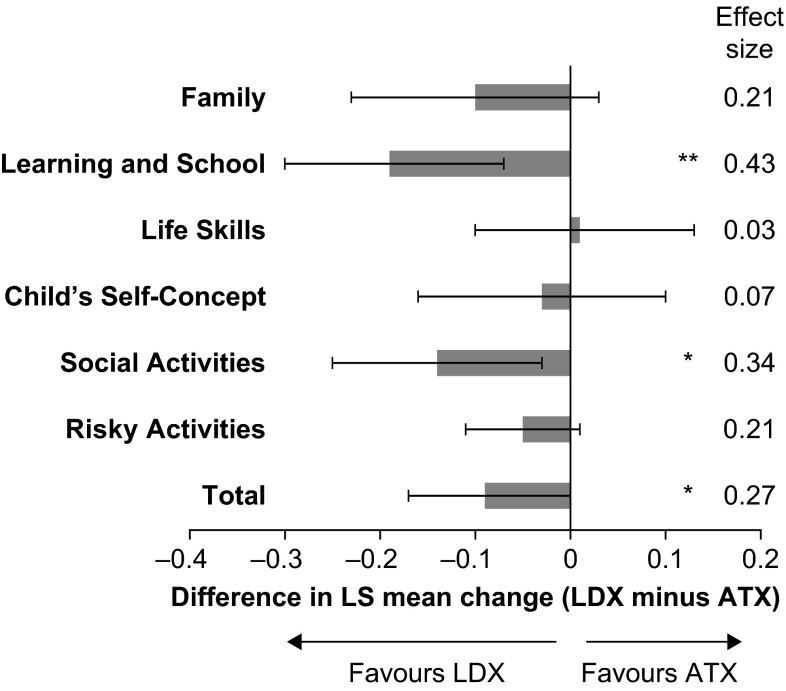
Difference between LDX and ATX groups in change in WFIRS-P scores from baseline to endpoint. *Histogram* shows the difference between treatment groups in LS mean change from baseline to endpoint. *Error bars* show 95 % confidence intervals. Endpoint was defined as the last on-treatment, post-baseline visit with a valid assessment. Effect size is the difference in LS mean change divided by root mean square error*. p* values are nominal and were not adjusted for multiple comparisons. *p < 0.05 and **p < 0.01 LDX versus ATX (pre-specified analysis). *ATX* atomoxetine, *LDX* lisdexamfetamine dimesylate, *LS* least-squares, *WFIRS-P* Weiss Functional Impairment Rating Scale-Parent Report

Compared with ATX treatment, LDX treatment was associated with numerically greater LS mean decreases from baseline to endpoint in WFIRS-P total score and scores in all domains except Life Skills. These differences between the LDX and ATX groups were statistically significant for total score [*p* = 0.046; effect size 0.27 (LDX vs ATX)] and the domains of Learning and School [*p* = 0.002; effect size 0.43 (LDX vs ATX)] and Social Activities [*p* = 0.014; effect size 0.34 (LDX vs ATX)] (Fig. 1).

### Change in WFIRS-P scores from baseline to week 9 and endpoint within treatment group

**Fig. 2:**
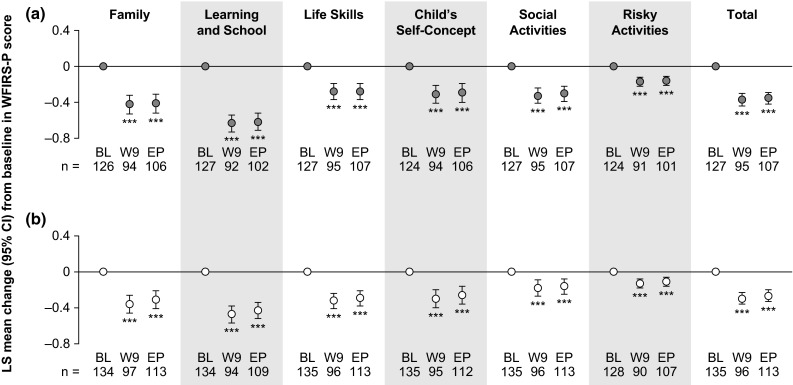
Change in WFIRS-P scores from baseline to week 9 and endpoint in (**a**) the LDX treatment group and (**b**) the ATX treatment group. The number of observations (*n*) shows the number of patients with a valid change from BL score at each time point. Endpoint was defined as the last on-treatment, post-baseline visit with a valid assessment. *p* values are nominal and were not adjusted for multiple comparisons. ***p < 0.001 versus baseline (ad hoc analysis). *ATX* atomoxetine, *BL* baseline, *CI* confidence interval, *EP* endpoint, *LDX* lisdexamfetamine dimesylate, *LS* least-squares, *n* number of observations, *W9* week 9, *WFIRS-P* Weiss Functional Impairment Rating Scale-Parent Report

Mean WFIRS-P total and domain scores at week 9 and endpoint are shown in Table 1. Both treatments were associated with statistically significant reductions from baseline (indicating improvement) in LS mean WFIRS-P total score at week 9 [LDX −0.37 (95 % CI −0.44, −0.30), ATX −0.30 (− 0.36, −0.23); both *p* < 0.001] and at endpoint [LDX −0.35 (95 % CI −0.42, −0.29), ATX −0.27 (− 0.33, −0.200); both *p* < 0.001] (Fig. 2). Reductions from baseline in LS mean scores in all WFIRS-P domains were also significant at week 9 and endpoint in both treatment groups (*p* < 0.001; Fig. 2). The greatest reductions were seen in the Learning and School domain at week 9 [LDX −0.63 (95 % CI −0.73, −0.54), ATX −0.47 (− 0.57, −0.38)] and at endpoint [LDX −0.62 (95 % CI −0.71, −0.52), ATX −0.43 (− 0.52, −0.34)].

In both the LDX and ATX treatment groups, the reductions from baseline to endpoint in LS mean WFIRS-P total score were greater than one-half the SD at baseline in the Full Analysis Set (0.247; Table S1). Within each individual domain, the reductions in LS mean score were greater than one-half the SD of the domain score at baseline (Table S1) in the domains of Family, Learning and School, and Life Skills in the LDX group and in the domains of Learning and School and Life Skills in the ATX group.

## Discussion

In this 9-week study, both LDX and ATX were associated with improvements in WFIRS-P total score and in scores across all six WFIRS-P domains in children and adolescents with ADHD who had experienced a clinically inadequate response to MPH therapy. The difference in these improvements between the LDX and ATX groups was statistically significant in favour of LDX for WFIRS-P total score and the Learning and School and the Social Activities domains, with effect sizes (LDX versus ATX) of 0.27, 0.43, and 0.34, respectively. These effect sizes are modest compared with the large effect size (0.924) of LDX versus placebo for improvement in WFIRS-P total score in a 7-week, randomized, double-blind, placebo-controlled study [5]. In the present head-to-head study, however, effect sizes are of LDX versus the active comparator, ATX, rather than versus placebo. Within each treatment group, the changes from baseline in WFIRS-P total score and some domain scores were greater than one-half SD for the population at baseline, suggesting that the observed improvements were clinically meaningful. However, the clinical relevance of the differences between active treatments in improvements in WFIRS-P scores remains to be established.

Depending on their age, patients with ADHD are more likely than their peers to underperform at school, to be unemployed or on a low income, to have accidents, to be arrested, to smoke or abuse substances, to become pregnant as teenagers, to contract sexually transmitted diseases, or to be divorced [1, 23]. Indeed, functional impairment is a diagnostic criterion for ADHD [2, 3, 43], and its impact on patients’ lives is commonly a major motivation for seeking treatment [37]. Therefore, effective treatments for ADHD should both improve symptoms and reduce the functional impairments that are associated with the disorder.

Relative to generic measures of function, disease-specific measures such as the WFIRS-P are believed to maximize sensitivity by focusing on particular areas of concern for patients with a given disorder and, as a result, may be especially useful for identifying and measuring treatment effects [21]. The sensitivity of the WFIRS-P to treatment-induced changes in functioning has been demonstrated in a limited number of published clinical trials [5, 32, 36, 39]; however, population-based normative data are not yet available [29]. Nevertheless, several unpublished industry-sponsored, government-funded or academically led interventional studies registered at clinicaltrials.gov specify the WFIRS-P as a secondary outcome measure, indicating its increasing acceptance as a measure of functional impairment outcomes in patients with ADHD [9–19].

Mean WFIRS-P total scores at baseline in the present study [LDX 0.95 (SD 0.474), ATX 0.91 (0.513)] were similar to those reported in two other published large-scale trials. In a European, 7-week, double-blind, randomized, placebo- and active-controlled study of LDX in 336 children and adolescents with ADHD, mean WFIRS-P total scores at baseline were in the range 1.01–1.10 (SD 0.437–0.456) across the three treatment groups [5]. In an international, 12-month, open-label, randomized study of ATX compared with ‘other standard therapy’ in 398 children and adolescents with ADHD, mean WFIRS-P scores at baseline were 1.02 (SD 0.475) and 0.96 (0.453) in the treatment groups, respectively [32]. All these baseline values exceed the optimal cut-off score (0.65) for differentiating children with and without ADHD, according to receiver operating characteristic curve analysis of a multicentre observational study [5, 29]. Furthermore, in all three studies, the highest mean WFIRS-P scores at baseline were observed in the Family domain and the Learning and School domain (also referred to as the Home domain and School domain [32]), indicating that profound impairments in these areas of day-to-day functioning are a consistent finding in clinical studies of children and adolescents with ADHD.

Despite differences in study design and patient inclusion/exclusion criteria, the levels of improvement in WFIRS-P total and domain scores in the LDX group of the present 9-week head-to-head study were similar to those observed in the previous 7-week placebo-controlled study of LDX [5]. In both studies, the largest improvements from baseline in the LDX group were observed in the Learning and School domain, followed by the Family domain, and the smallest improvements were seen in the Life Skills and the Risky Activities domains. In the placebo-controlled study, the improvements from baseline were statistically significantly superior to placebo for WFIRS-P total score and the domains of Learning and School, Family, Social Activities, and Risky Activities. Some patients from the European placebo-controlled study, together with additional US patients, took part in a follow-up study of the long-term maintenance of efficacy of LDX [24], which also included the WFIRS-P as a secondary efficacy measure. In the open-label period of at least 26-week LDX treatment, WFIRS-P scores improved in all domains and in total [4]. In the subsequent 6-week, double-blind, placebo-controlled, randomized-withdrawal period, WFIRS-P scores deteriorated in the placebo group in all domains and in total, and the difference between the LDX and placebo groups was statistically significant for WFIRS-P total score and the domains of Family, Learning and School, and Risky Activities [4].

In the present study, the largest changes from baseline in the ATX group were observed in the Family domain and the Learning and School domain, as was also the case in the LDX group. These domains also saw the largest changes from baseline in both treatment arms of the 12-month open-label study comparing ATX with ‘other standard therapy’ (mainly MPH) [32] and in the osmotic-release oral system MPH (OROS-MPH) reference arm of the 7-week European, placebo-controlled study of LDX [5]. This reference arm was included to validate the study design and contextualize the results; statistical comparisons of OROS-MPH versus LDX were not pre-specified [22].

Taken together, the available data therefore suggest that the Learning and School and the Family domains of the WFIRS-P are most responsive to therapy, as well as most affected at baseline. This does not necessarily mean, however, that the effects of different classes of ADHD medication on different domains of functional impairment are equivalent. For example, in the present study, LDX was statistically significantly superior to ATX in total score and in the Social Activities and the Learning and School domains, but not in other domains. Also, in the 7-week, European, placebo-controlled study of LDX, the effect sizes for WFIRS-P total score of LDX versus placebo and of the OROS-MPH reference treatment versus placebo were both large (0.924 and 0.772, respectively), but the placebo-adjusted effects of the two medications were not identical across the six WFIRS-P domains [5] (no post hoc statistical analysis comparing LDX versus OROS-MPH has been published for these outcomes, in contrast to the primary efficacy outcome [38]). The potential implications of these findings and the present results for individualized patient management remain to be established.

Although the present study did not investigate the time course of changes in WFIRS-P scores, evidence from previous studies suggests that the rate of response to treatment may differ across WFIRS-P domains. First, within both the LDX arm and OROS-MPH reference arm of the European, placebo-controlled study of LDX, some domains did not show improvement from baseline until week 7, whereas improvement from baseline in others was evident at week 4 [5]. Secondly, most, but not all, of the improvement from baseline in WFIRS-P scores in the open-label period of the subsequent randomized-withdrawal study occurred before week 8 [4]. Thirdly, in the long-term, open-label comparison of ATX with ‘other standard therapy’, the only statistically significant difference in WFIRS-P scores between groups was in favour of ‘other standard therapy’ in the Learning and School domain at 6 months (*p* < 0.05); this difference was not evident after 12 months of treatment [32]. These observations suggest that some domains of functional impairment may be more likely than other aspects of ADHD to respond to pharmacotherapy over a longer treatment period than was used in the present study.

The associations between ADHD symptoms, functional impairments, and health-related quality of life (HRQoL) have yet to be fully understood. HRQoL instruments measure a patient’s (or a parent’s) subjective perception of the impact of health status on their (or their child’s) physical, psychological, and social well-being [26]. Studies investigating the correlations between ADHD symptom severity ratings and HRQoL scores have revealed statistically significant, but only moderately strong, correlations between the two measures (*r* = 0.2–0.6), supporting the view that symptoms and HRQoL represent related but distinct constructs [26]. There are, however, few data available to establish the interactions between functional impairment and either ADHD symptoms or HRQoL. Analyses have indicated a statistically significant, but imperfect, correlation between functioning and HRQoL, suggesting that these too are related but distinct constructs [20]. This is consistent with the observation in the present study that the effect sizes of LDX versus ATX for WFIRS-P scores (total score 0.27, Learning and School 0.43, Social Activities 0.34, all *p* < 0.05) were smaller than for the previously reported ADHD-RS-IV symptomatological outcome measure (0.56; *p* < 0.001) [27]. Effect sizes for LDX and OROS-MPH versus placebo were also larger for ADHD-RS-IV total score than for WFIRS-P scores in the previous 7-week, European, placebo-controlled study [5, 22]. Further studies and analyses are required to increase the understanding of the interrelationships between ADHD symptoms, functional impairment, and HRQoL.

The safety outcomes of the present study have been published previously and were consistent with those of earlier clinical trials [27]. In summary, treatment-emergent adverse events were reported by similar proportions of patients in each treatment group (LDX 71.9 %, ATX 70.9 %). [27] Weight loss was larger in the LDX group than in the ATX group, and the proportion of patients who met the outlier criterion for high pulse rate (≥100 beats per minute) was higher in the ATX group than in the LDX group [27]. With these exceptions, the changes in mean vital signs and electrocardiogram parameters, the proportions of patients meeting other outlier criteria, and the frequency of potentially clinically important vital sign observations were all similar for LDX and ATX treatment [27].

Strengths of the present study include the sample size, the head-to-head, randomized, double-blind, dose-optimized design, and the geographically diverse patient population. Patients were required to have experienced an inadequate response to MPH therapy, making the present findings particularly relevant in European countries in which LDX is approved for the treatment of children and adolescents when response to previous MPH treatment is considered clinically inadequate by the supervising specialist. Although the definition of inadequate response to MPH therapy was broad, lack of efficacy was the most common reason given for an inadequate response.

An important limitation of the study is the relatively short duration of treatment, which precludes evaluation of the long-term effects of treatment on functional impairment. The observation that it may take 12 or more weeks for ATX to reach its maximum effect on ADHD symptoms [42] suggests the possibility that the 9-week duration of the study may have led to an underestimation of the efficacy of ATX. Improvements in functioning may occur over a longer treatment period than relief of symptoms, potentially also leading to underestimation of the effects of treatment on functional impairment as a secondary outcome in the present study. Furthermore, although the dose-titration schedules of both treatments followed current guidelines, twice-daily ATX and/or higher doses of ATX may have been more effective than the once-daily dosing required in the present study [35]. Finally, the possibility cannot be excluded that patients’ experience of MPH treatment before enrolment may have led to preconceptions regarding the ADHD medications under investigation (perhaps relating to their onset or duration of efficacy or tolerability), which might have influenced functional outcomes in this double-blind study.

In conclusion, this study has shown that both LDX and ATX treatment can improve functioning, as measured by the WFIRS-P, in children and adolescents with ADHD who have experienced a clinically inadequate response to MPH (as judged by investigators). Improvements overall and in certain domains were statistically significantly greater in magnitude with LDX treatment than with ATX treatment, within the time frame of the study. These findings may aid clinicians when developing treatment plans to address the functional impairments associated with ADHD.

## Electronic supplementary material

Supplementary material 1 (PDF 841 kb)

Supplementary material 2 (DOCX 127 kb)
