# Supplementary figures and images for: Functional outcomes from a head-to-head, randomized, double-blind trial of lisdexamfetamine dimesylate and atomoxetine in children and adolescents with attention-deficit/hyperactivity disorder and an inadequate response to methylphenidate
Source: Eur Child Adolesc Psychiatry. 2015 May 22;25:141–9. doi: 10.1007/s00787-015-0718-0 (PMC4735245; doi:10.1007/s00787-015-0718-0)

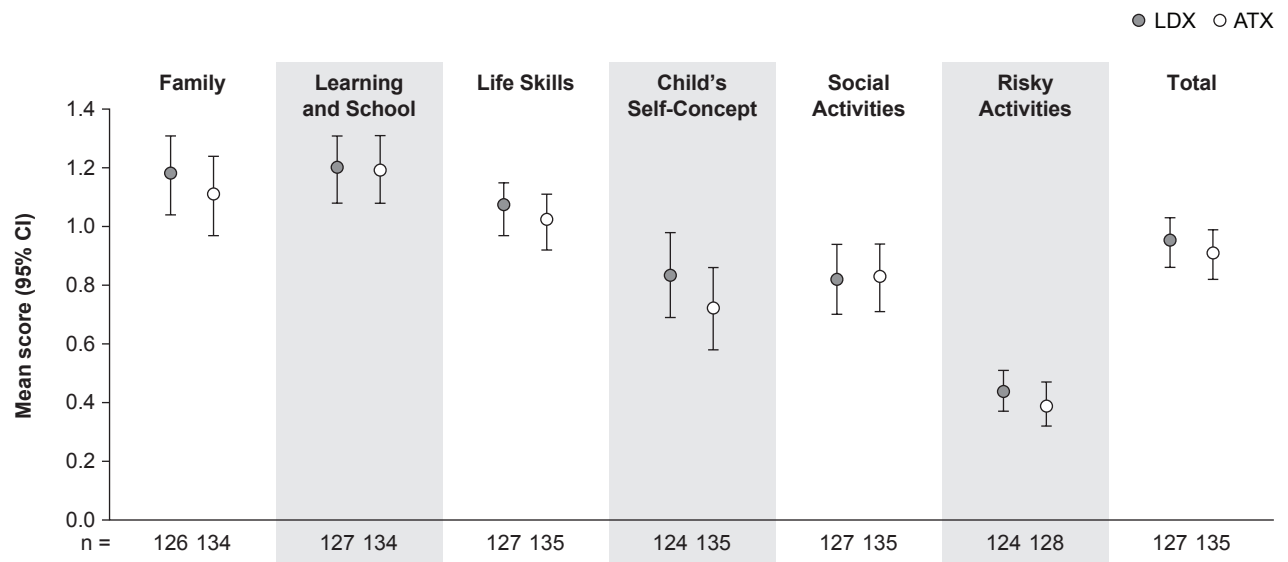

Supplement: Supplementary file 1 — Supplementary material 1 (PDF 841 kb) [file 787_2015_718_MOESM1_ESM.pdf]
