# Supplementary material for: Functional outcomes from a head-to-head, randomized, double-blind trial of lisdexamfetamine dimesylate and atomoxetine in children and adolescents with attention-deficit/hyperactivity disorder and an inadequate response to methylphenidate
Source: Eur Child Adolesc Psychiatry. 2015 May 22;25:141–9. doi: 10.1007/s00787-015-0718-0 (PMC4735245; doi:10.1007/s00787-015-0718-0)
Supplement: Supplementary file 2 — Supplementary material 2 (DOCX 127 kb) [file 787_2015_718_MOESM2_ESM.docx]

# Supplementary Figure

### **Table S1. WFIRS-P total and domain scores at baseline in all patients in the Full Analysis Set (n = 262)**

|  | Number of  observations | Mean, SD | 0.5 SD |
| --- | --- | --- | --- |
| Family | 260 | 1.15 (0.777) | 0.389 |
| Learning and School | 261 | 1.20 (0.663) | 0.332 |
| Life Skills | 262 | 1.05 (0.535) | 0.268 |
| Child’s Self-Concept | 259 | 0.77 (0.820) | 0.410 |
| Social Activities | 262 | 0.82 (0.682) | 0.341 |
| Risky Activities | 252 | 0.42 (0.406) | 0.203 |
| **Total** | 262 | 0.93 (0.494) | 0.247 |
|  |  |  |  |

Higher scores indicate greater impairment. Scores are based on observed values. Numbers of observations depended on domain.

SD, standard deviation; WFIRS-P, Weiss Functional Impairment Rating Scale-Parent Report

### **Fig S1.** WFIRS-P total and domain scores at baseline


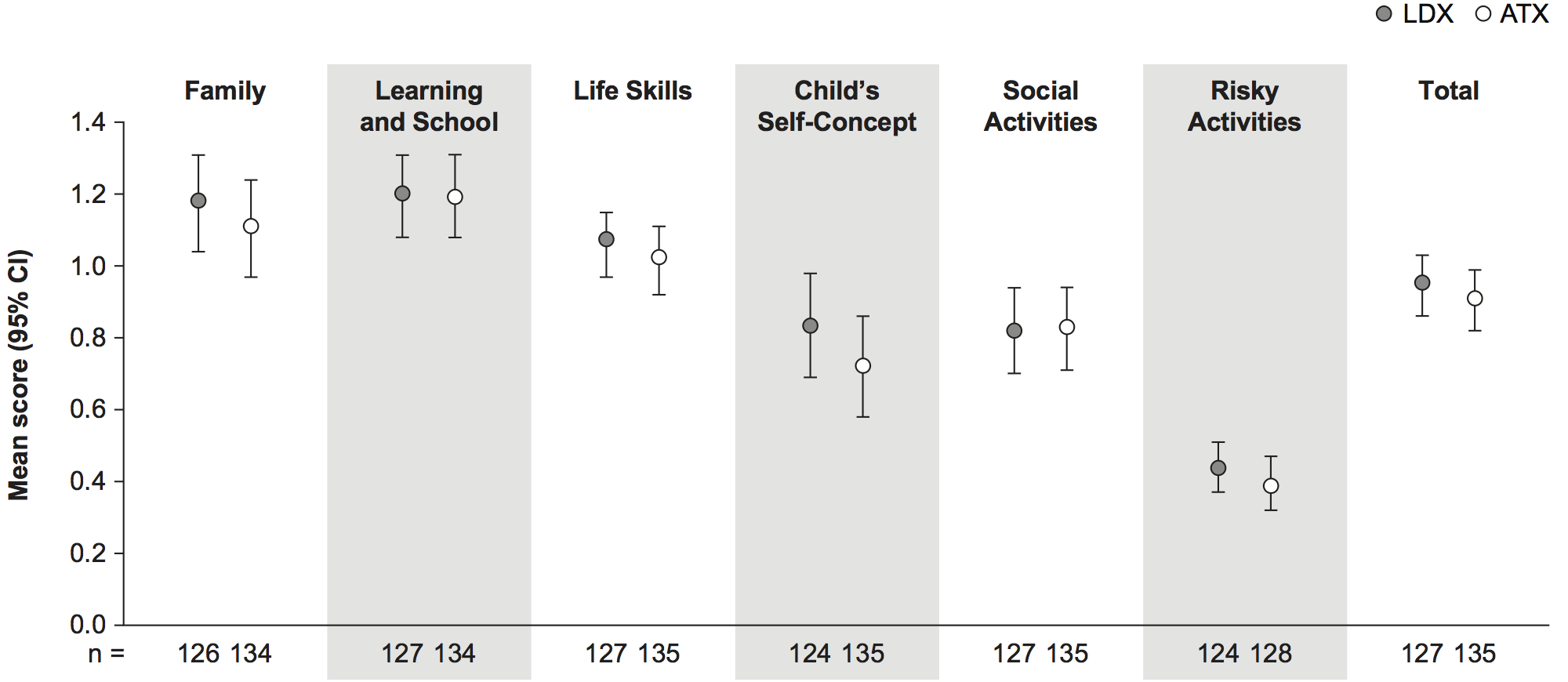


The number of observations (n) shows the number of patients with a valid score at baseline. Baseline scores were not analysed statistically. A statistically significant difference (α = 0.05) between treatment groups for each domain or for total score would not be detected in a two-tailed *t* test when a treatment group mean value lies within the 95% confidence interval (CI) of the other treatment group mean. Higher scores indicate greater impairment.

ATX, atomoxetine; CI, confidence interval; LDX, lisdexamfetamine dimesylate; n, number of observations; WFIRS-P, Weiss Functional Impairment Rating Scale-Parent Report.
